# Outer membrane vesicles of *Porphyromonas gingivalis*: recent advances in pathogenicity and associated mechanisms

**DOI:** 10.3389/fmicb.2025.1555868

**Published:** 2025-04-01

**Authors:** Zixiang Wu, Wei Long, Yi Yin, Biyun Tan, Chenyu Liu, Hongqiao Li, Song Ge

**Affiliations:** School and Hospital of Stomatology, Zunyi Medical University, Zunyi, Guizhou, China

**Keywords:** periodontitis, *Porphyromonas gingivalis*, outer membrane vesicles, virulence factors, pathogenesis, systemic diseases

## Abstract

Periodontitis is a chronic infectious inflammatory disease primarily caused by periodontal pathogenic bacteria, which poses a significant threat to human health. The pathogenic mechanisms associated with *Porphyromonas gingivalis* (*P. gingivalis*), a principal causative agent of periodontitis, are particularly complex and warrant thorough investigation. The extensive array of virulence factors released by this bacterium during its growth and pathogenesis not only inflicts localized damage to periodontal tissues but is also intricately linked to the development of systemic diseases through various mechanisms. The outer membrane vesicles (OMVs) produced by *P. gingivalis* play a key role in this process. These OMVs serve as important mediators of communication between bacteria and host cells and other bacteria, carrying and delivering virulence factors to host cells and distant tissues, thereby damaging host cells and exacerbating inflammatory responses. The ability of these OMVs to disseminate and deliver bacterial virulence factors allows *P. gingivalis* to play a pathogenic role far beyond the confines of the periodontal tissue and has been closely associated with the development of a variety of systemic diseases such as cardiovascular disease, Alzheimer’s disease, rheumatoid arthritis, diabetes mellitus, non-alcoholic hepatitis, and cancer. In view of this, it is of great pathophysiological and clinical significance to deeply investigate its pathogenic role and related mechanisms. This will not only help to better understand the pathogenesis of periodontitis and its related systemic diseases but also provide new ideas and more effective and precise strategies for the early diagnosis, prevention, and treatment of these diseases. However, the current research in this field is still insufficient and in-depth, and many key issues and mechanisms need to be further elucidated. This article summarizes the recent research progress on the role of *P. gingivalis* OMVs (*P. g*-OMVs) in related diseases, with the aim of providing a theoretical basis and direction for future research and revealing the pathogenic mechanism of *P. g*-OMVs more comprehensively.

## 1 Introduction

Various cell types, such as immune cells, stem cells, endothelial cells (ECs), neuronal cells, etc., as well as bacteria (including Gram-negative and Gram-positive bacteria) and fungi, have the ability to release extracellular vesicles (EVs). Based on cellular origin, biological function, size, biogenesis, and biochemical composition, EVs can be categorized into three main subclasses: extracellular vesicles, microvesicles, and apoptotic vesicles. Among them, Gram-negative bacteria produce EVs, which are also known as outer membrane vesicles (OMVs) (Beveridge, 1999; Johnston et al., 2023). In the 1960s, S. N. Chatterjee and J. Das first observed OMVs in the cell walls of Gram-negative bacteria by electron microscopy (Chatterjee and Das, 1967). With the deepening understanding of OMVs, it was found that OMVs have important biological functions, such as delivering virulence factors to host cells, inducing host immune responses, promoting bacterial biofilm formation, maintaining biofilm homeostasis, and participating in the bacterial stress response (Gui et al., 2016; Chatterjee and Das, 1967). In addition, OMVs can utilize the advantages they possess to expand the infection range of parental bacteria, enhance their pathogenicity, and promote the immune inflammatory response of the host organism. This suggests that OMVs may play an important role in bacterial pathogenesis and in inducing the development of systemic diseases (Santos et al., 2018; Olovo et al., 2024).

*Porphyromonas gingivalis* (*P. gingivalis*) is a Gram-negative, specialized anaerobic bacterium that, as a key causative agent of periodontitis, produces a large number of virulence factors that attack host cells during its growth, including hemagglutinin, lipopolysaccharide (LPS), gingipains, and fimbriae, among others (Williams and Holt, 1985). *P. gingivalis* and the virulence factors it produces not only cause periodontal lesions leading to tooth loss but also induce local production of pro-inflammatory cytokines, prostaglandins, and matrix metalloproteinases (MMPs) throughout the pathogenic process, which can diffuse into the systemic circulation via the epithelium of the inner wall of ulcerated periodontal pockets (Zheng et al., 2021). Thus, in addition to its important pathogenic role in the periodontitis process, *P. gingivalis* is also closely associated with the development of systemic diseases (Zhou et al., 2021).

All *P. gingivalis* strains to date have been found to produce OMVs and possess the main biological properties of the parental bacteria (Zhang et al., 2021). *P. g*-OMVs concentrate and carry a large number of virulence factors of the parental bacteria, including LPS, gingipains, and peptidylarginine deiminase (PPAD), making them play important pathogenic roles in the development of periodontitis and systemic diseases (Grenier and Mayrand, 1987). Therefore, modulating or inhibiting the formation of *P. g*-OMVs may be one of the therapeutic targets to reduce the virulence of *P. gingivalis*. More literature describes the role of *P. gingivalis* in the development of related diseases and the molecular mechanisms involved in the signaling of disease-related cells, and so on (Xu et al., 2020). Therefore, this paper provides a more comprehensive review on the occurrence and regulation of *P. g*-OMVs, their components and pathogenic roles, as well as the roles of *P. g*-OMVs in the association of periodontitis and systemic diseases and related mechanisms, with the aim of providing new research ideas and strategies for the prevention and treatment of periodontitis and systemic diseases.

## 2 Biogenesis and regulation of *P. g*-OMVs

**Figure 1:**
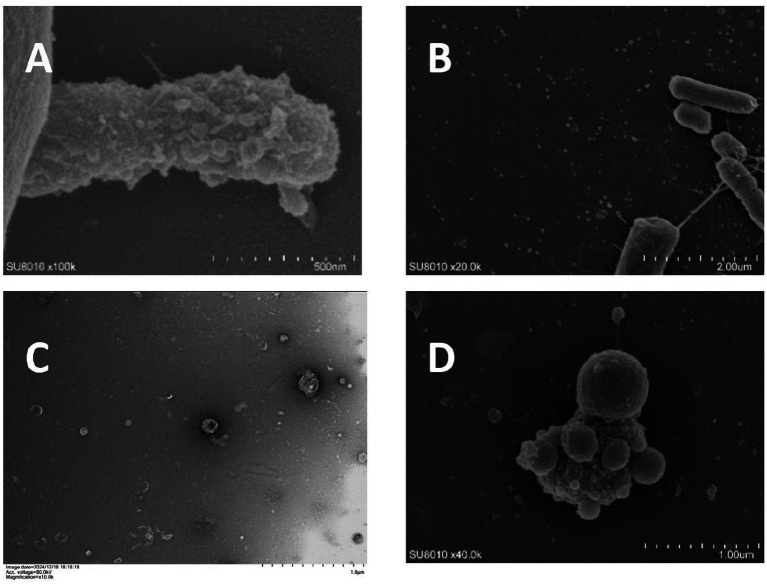
Electron microscope image of *Porphyromonas g*-OMVs (unpublished results). **(A)** Scanning electron micrograph showing OMVs blebbing from the surface of a *P. gingivalis* cell. **(B)** Scanning electron microscope showing a large number of OMVs scattered around *P. gingivalis*. **(C)** Transmission electron microscopy showing a large number of similar spherical *P. g*-OMVs with diameters ranging from 20 to 250 nm. **(D)** Scanning electron microscope showing a large number of OMVs on and around the surface of *P. gingivalis* under the effect of 2-fold MIC metronidazole concentration.

The envelope of *P. gingivalis* is comprised of an inner membrane formed by phospholipid bilayers, an outer membrane, and a peptidoglycan layer situated between the two membranes. The interstitial space between the inner and outer membranes is referred to as the periplasm, which contains proteins, peptidoglycans, and various other components (Toyofuku et al., 2019). *P. g*-OMVs are nanospherical structures generated by the budding of the outer membranes, with diameters ranging from approximately 50 to 250 nanometers (Figures 1A–C). It has been documented that Gram-negative bacteria can produce OMVs through multiple pathways, and the mechanisms underlying OMVs formation can differ both among distinct bacterial species and within the same species (Gui et al., 2016).

**Figure 2:**
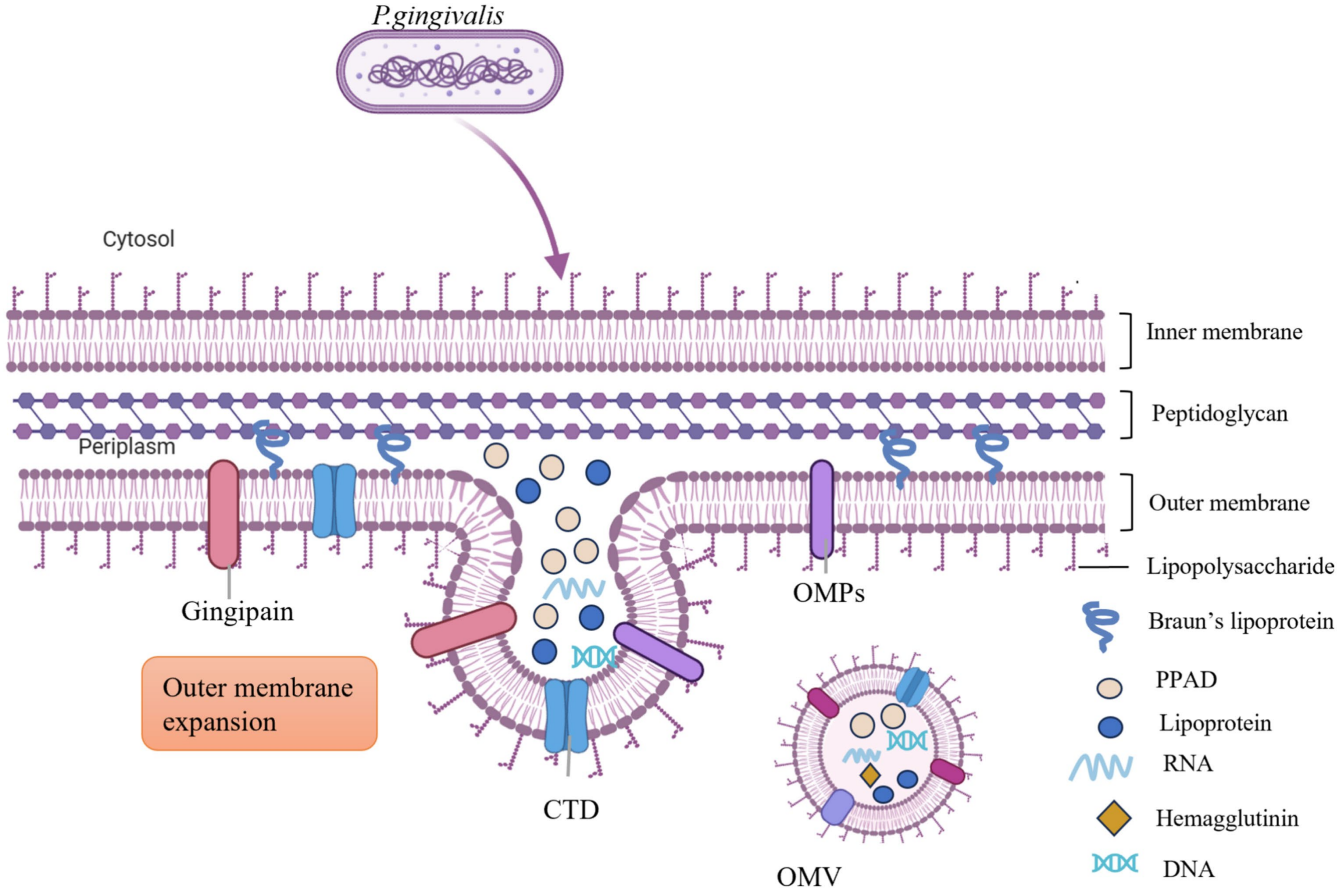
The biogenesis process of *P. g*-OMVs involves the accumulation of phospholipids in the outer membrane, which facilitates the outward expansion of this membrane. The presence of both positive and negative curvature on the outer membrane encourages further enrichment of phospholipids and supports the growth of the outer membrane. Additionally, the accumulation of misfolded proteins and molecules, including LPS and peptidoglycan fragments within the periplasm, generates pressure on the outer membrane, leading to its outward expansion. This process also encapsulates a significant number of virulence factors from the parental cells, such as LPS, gingipains, PPAD, and miRNA, among others. OMPs, outer membrane proteins; CTD, C-terminal regulatory structural domain; DNA, deoxyribonucleic acid; RNA, ribonucleic acid; PPAD, peptidylarginine deiminase; LPS, lipopolysaccharide.

The biogenesis of *P. g*-OMVs remains incompletely understood; however, several models have been proposed to elucidate this process (Figure 2). Initially, the VacJ/Yrb genes are known to regulate lipid homeostasis within the outer membrane (Toyofuku et al., 2019). Collectively, the VacJ and Yrb genes form the Maintenance of Lipid Asymmetry (Mla) system, which is crucial for preserving lipid asymmetry in the outer membrane of *P. gingivalis*. VacJ encodes transmembrane proteins that facilitate the transport of phospholipids within the inner leaflet of the outer membrane, enabling the recycling of excess phospholipids from the outer membrane back to the inner membrane through reverse transport. The Yrb complex, located in both the periplasm and the inner membrane, collaborates with VacJ to function as a phospholipid “reverse transporter expression of either the VacJ or Yrb genes is diminished or absent, the recycling of phospholipids becomes inefficient, resulting in an abnormal accumulation of phospholipids in the inner leaflet of the outer membrane. This accumulation disrupts the natural lipid asymmetry of the outer membrane, leading to localized asymmetric dilation and the formation of outwardly expanding vesicular structures. Additionally, membrane curvature facilitates the enrichment of phospholipids and promotes vesicle outgrowth (Gui et al., 2016; Rocha et al., 2021). The excessive accumulation of phospholipids in the outer membrane alters the physical properties of the membrane lipids. Regions exhibiting positive curvature (convexity) and negative curvature (concavity) contribute to the further enrichment of phospholipid molecules in areas of high tension, thereby creating localized membrane curvature. This curvature effect directly supports the outward budding of the outer membrane, ultimately resulting in the release of OMVs. Moreover, periplasmic pressure and the accumulation of molecules further promote the expansion of the outer membrane (Juodeikis and Carding, 2022). The buildup of misfolded proteins and molecules, such as LPS and peptidoglycan fragments, within the periplasm exerts compressive forces that induce the outward expansion of the outer membrane of *P. gingivalis*, thereby facilitating the formation of OMVs. Additionally, the disruption of the connection between the outer membrane and the peptidoglycan layer is essential for OMV formation. The outer membrane is covalently linked to the underlying peptidoglycan layer via lipoproteins, which are critical for maintaining structural stability. The formation of OMVs necessitates localized disruption of this connection. The degradation of the peptidoglycan layer by glycosidases or peptidases, along with the physical tension experienced during membrane expansion, can compromise the outer membrane-peptidoglycan layer connection, allowing the outer membrane to detach from the cell wall and facilitate vesicle release (Juodeikis and Carding, 2022).

The biogenesis of *P. g*-OMVs is influenced by a variety of factors. Previous research has indicated that elements such as the production levels of autolysin, the genotype of fimbriae protein A (FimA), and the presence of gingipains (including arginine gingipains [Rgps] and lysine gingipains [Kgp]) significantly affect the biogenesis of *P. g*-OMVs (Juodeikis and Carding, 2022; Mantri et al., 2015). Recent studies have identified additional factors that impact the production of *P. g*-OMVs. Outer membrane protein A (OmpA) possesses a unique structure and characteristics that enhance the support and stability of the outer membrane by cross-linking with the peptidoglycan layer, thereby maintaining the integrity and normal architecture of the outer membrane (Iwami et al., 2007). The distribution and localization of OmpA within the cell membrane may influence membrane bending and deformation, which in turn promotes the bulging of localized regions of the outer membrane and is implicated in the regulation of OMV formation. Iwami et al. (2007) observed that the surface of the *P. gingivalis* OmpA mutant and its surrounding medium exhibited a significantly higher quantity of OMVs compared to the control group. LPS is composed of lipid A, a polysaccharide core, and a long chain of polysaccharides known as the O-antigen. This structure is involved in the selective sorting of outer membrane proteins (OMPs) into *P. g*-OMVs, and its phosphorylation status may also influence the stability of the outer membrane of *P. gingivalis*, potentially affecting the formation process of *P. g*-OMVs (Haurat et al., 2011). Lipid A is a critical component of the bacterial outer membrane, and its structural and modification status can impact the stability and curvature of the outer membrane, which is essential for regulating OMV formation. Alaei et al. (2024) demonstrated that lipid A C4’-phosphatase (lpxF) deletion mutant strains of *P. gingivalis*, as well as strains with inactivating point mutations in the LpxF active site, exhibited significantly reduced production of *P. g*-OMVs compared to wild-type strains. This finding suggests that the removal of the C4’-phosphate group from lipid A is necessary for the formation of *P. g*-OMVs. LpxF is an enzyme that plays a vital role in the biosynthesis of bacterial lipid A. The removal of the C4’-phosphate group by LpxF alters the charge distribution, spatial structure, and phosphorylation state of lipid A, leading to increased hydrophobicity and a relative decrease in hydrophilicity, which subsequently affects the arrangement and stability of lipid A in cell membranes. This alteration impacts the flexibility and fluidity of the outer membrane, as well as increases its curvature, thereby facilitating the formation of *P. g*-OMVs. Similarly, Rangarajan et al. (2017) provided further evidence that modifications to the structure of lipid A may influence the stability of the outer membrane of *P. gingivalis* and thus regulate the formation of *P. g*-OMVs. Lipopolysaccharide transport protein O (LptO) (PG0027) is a key protein that regulates lipid A 1-phosphatase activity in *P. gingivalis* strain W50. Through its 1-phosphatase activity, LptO can remove the phosphoryl group at the 1-position of the lipid A molecule, thereby altering its structure. This suggests that the modification of lipid A by LptO may represent a potential mechanism for regulating the formation of *P. g*-OMVs. In contrast, Alaei et al. (2024) found that strains lacking lipid A C1-phosphatase (lpxE) or lipid A deacetylase (PGN_1123; lpxZ) genes did not exhibit significant changes in the number of *P. g*-OMVs compared to wild-type strains. This indicates that C1-phosphate modification and deacylation of lipid A have a lesser impact on the biogenesis of *P. g*-OMVs. Furthermore, this study revealed that the structure of lipid A influences the functionality of *P. g*-OMVs; specifically, *P. g*-OMVs produced by ΔlpxF and ΔlpxZ strains, which possess a pentanoylated form of lipid A, were found to strongly activate Toll-like receptor 4 (TLR4) in the host. Conversely, *P. g*-OMVs produced by wild-type and ΔlpxE strains, which predominantly exhibit a tetraacylated form of lipid A, were unable to activate TLR4. This suggests that the degree of acylation of lipid A is a determining factor in the ability of *P. g*-OMVs to interact with host TLR4. The UDP-galactose-4-epimerase gene (GalE) is a widely distributed gene in bacteria that is involved in galactose metabolism, and the enzyme it encodes plays a crucial role in LPS synthesis. Abnormal expression of the GalE gene may alter the structure and quantity of synthesized LPS, thereby affecting the formation of OMVs. Nakao et al. (2011) found that *P. gingivalis* GalE mutant strains produced significantly fewer *P. g*-OMVs than their wild-type counterparts. A recent study by Rothenberger et al. (2024) identified a *P. g*-OMVs-targeted mycobacterial-like lipoprotein (PG1881), which exhibits high structural similarity to FimA and Major Fimbrial Protein 1 (Mfa1) produced by *P. gingivalis*. It was proposed that PG1881 may play a role in the emergence and formation of *P. g*-OMVs from the cell membrane of *P. gingivalis*. To effectively colonize the gingival sulcus of teeth, *P. gingivalis* must adapt to environmental stresses, and the expression of its environmental stress-responsive genes is regulated by the extracellular function (ECF) *σ*-factor (Fujise et al., 2017). Fujise et al. (2017) reported that *P. gingivalis* SigP mutant (one of the ECF σ-factor genes) produced a greater number of OMVs on its surface compared to the wild-type strain PGN-0450 and the sigH mutant. Additionally, this study found that SigP is also involved in the activity of gingipains, self-aggregation, hemagglutination activity, and susceptibility to antimicrobial agents in *P. gingivalis* (Fujise et al., 2017). PPAD is an enzyme secreted by *P. gingivalis* that facilitates bacterial surface translocation. Vermilyea et al. (2021) found that the absence of PPAD resulted in a significant reduction in *P. g*-OMV production. Furthermore, the growth stage of the bacteria also influences the production of *P. g*-OMVs. The study conducted by Mao et al. (2023) indicated that the quantity of *P. g*-OMVs increases with prolonged incubation time, with both the amount of *P. g*-OMVs and its protein concentration peaking during the stabilization growth phase of the bacteria. Similarly, Zhang et al. (2022) reported that the ratio of *P. g*-OMVs to bacteria in cultures at the stabilization growth stage was approximately 2000:1.

In recent years, research has indicated that various environmental factors influencing bacterial growth may play a significant role in the regulation of OMVs formation. These factors include antibiotics, environmental pH levels, temperature, nutrient concentrations, and oxidative stress. Numerous studies have demonstrated that various antibiotics, administered at different concentrations, can enhance the production of OMVs in bacteria. For instance, Kadurugamuwa and Beveridge (1995) reported that exposure to four times the minimum inhibitory concentration (MIC) of gentamicin resulted in an approximate threefold increase in the release of OMVs from *Pseudomonas aeruginosa* (*P. aeruginosa*). Additionally, sub-MIC levels of ciprofloxacin significantly elevated the production of OMVs by *Escherichia coli* (*E. coli*), with increases observed up to 250-fold (Bauwens et al., 2017). The impact of bacterial OMVs has important implications. On the one hand, increased production of OMVs may enhance the ability of bacteria to spread drug resistance, such as by carrying more resistant genes for horizontal transfer and spreading them within bacterial populations; on the other hand, changes in OMVs may also affect their pathogenicity and ecological adaptability. For example, Bauwens et al. (2017) found that ciprofloxacin not only increased the production of OMVs, but also up-regulated Shiga toxin 2a associated with OMVs, which may exacerbate the clinical consequences of infections caused by Shiga toxin-producing *E. coli*. There are no definitive studies reporting that antibiotics affect the production and pathogenicity of *P. g*-OMVs (Figure 1D). However, this is a very worthwhile direction for research. An in-depth understanding of how antibiotics affect the production and pathogenicity of *P. g*-OMVs and their mechanisms is important for understanding the development of drug resistance in *P. gingivalis*, the mechanisms of pathogenicity, and the development of new antimicrobial strategies. Johnston et al. (2023) found that a decrease in the pH in the surrounding environment led to an increase in the production of *Helicobacter pylori* (*H. pylori*) OMVs. Different levels of pH were also found to affect the composition and proteome of OMVs, which in turn may affect their subsequent biological functions. However, the effect of pH of the growth environment on the production of *P. g*-OMVs and their composition remains to be further determined.

## 3 Pathogenic components of *P. g*-OMVs and mechanisms

**Figure 3:**
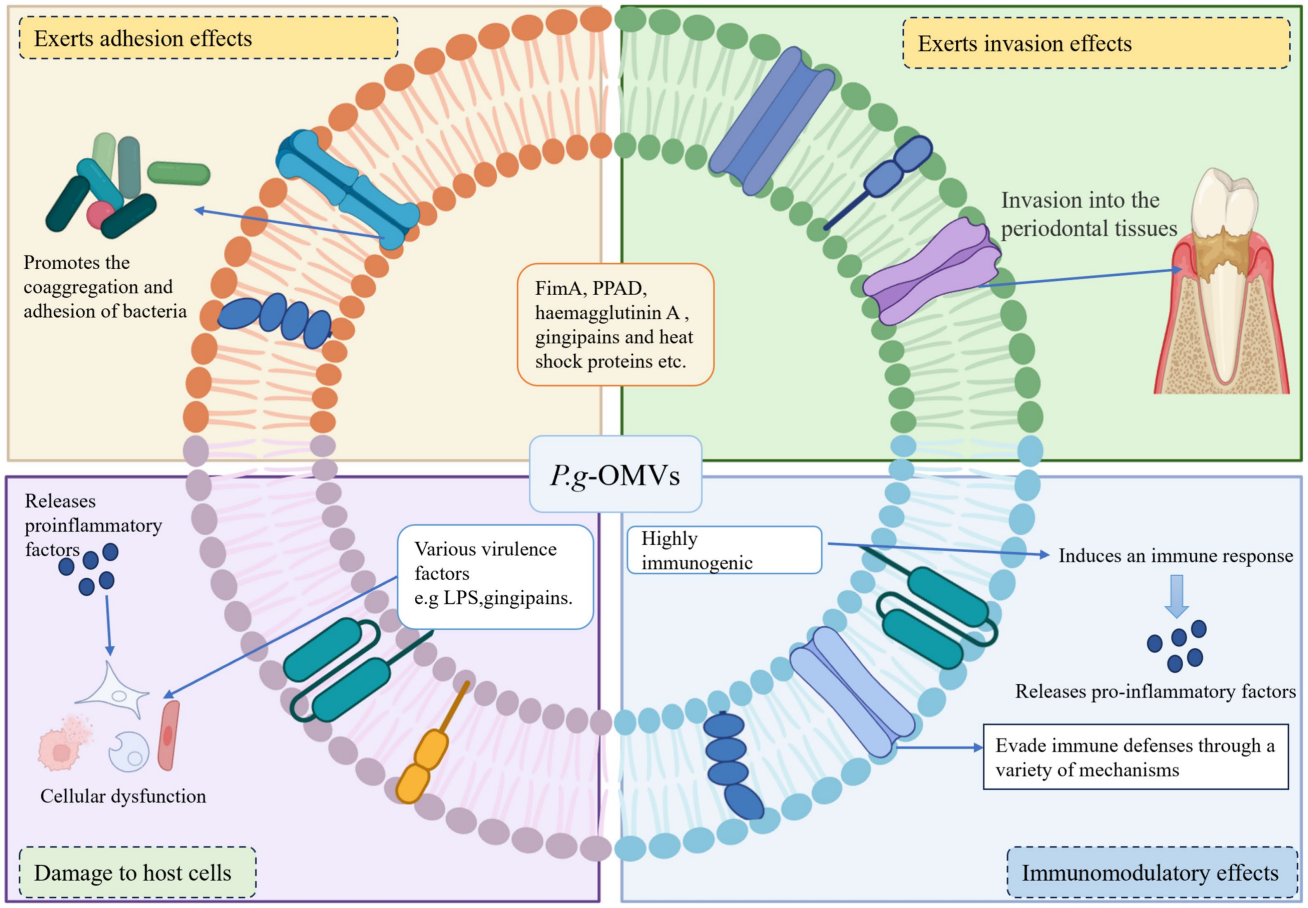
Pathogenic mechanism of *P. g*-OMVs. (1) Playing an adhesive role: *P. g*-OMVs promote bacterial adhesion between non-coaggregating bacteria by encapsulating adhesion molecules of parental bacteria, such as FimA, PPAD, haemagglutinin A, gingipains, and heat shock proteins. (2) Playing an invasive role: *P. g*-OMVs can interact with surface receptors on host cells by encapsulating membrane proteins such as FimA, haemagglutinin A, and heat shock proteins, virulence factors such as surface-enriched gingipains and adhesins, as well as fimbriae, which can contribute to the invasive effect of *P. g*-OMVs, and also affect the ability of other bacteria to attach to and invade host cells. (3) Immunomodulatory effect: *P. g*-OMVs are highly immunogenic and can interact with immune cells, thereby exerting immunomodulatory effects. In addition, *P. g*-OMVs can also exert their pathogenic effects by supporting the evasion of host immune defense mechanisms. (4) Damage to the host cells: The invasion of *P. g*-OMVs into host cells can lead to host cell dysfunction by affecting cell proliferation and migration, causing cell death or other forms of cell death, and inducing a severe inflammatory response in the cells, which in turn can play a role in damaging host cells. FimA, fimbriae protein A; PPAD, peptidylarginine deiminase; LPS, lipopolysaccharide.

The pathogenic effects of *P. g*-OMVs are significantly influenced by their protein and nucleic acid content (Veith et al., 2022). Veith et al. (2014) characterized the protein composition of *P. g*-OMVs through mass spectrometry, identifying a total of 151 proteins, which include 127 outer membrane proteins and 24 periplasmic proteins. These proteins are essential for the formation, stability, and functionality of *P. g*-OMVs. The proteome of *P. g*-OMVs is predominantly composed of proteins associated with the type IX secretion system (T9SS) (Veith et al., 2022). The T9SS facilitates the loading of a high density of protein virulence factors onto the outer membrane of *P. gingivalis*, including gingipains, adhesins, hemolysins, iron uptake proteins, and endocannabinoids (Veith et al., 2014; Veith et al., 2022). These factors are differentially packaged in response to environmental conditions and are released into *P. g*-OMVs in a structurally stable and active form (Veith et al., 2022; Veith et al., 2018). This dynamic composition of virulence factors indicates that *P. g*-OMVs can adapt to varying growth conditions. Furthermore, this adaptability enables *P. g*-OMVs to play a significant role in host tissue destruction, dysregulation of the immune response, internalization, and the capture of (micro)nutrients. Further analysis revealed that OmpA, gingipains (including Rgps and Kgp), and LPS were found to be highly abundant in *P. g*-OMVs. Gingipains, a class of proteolytic enzymes synthesized by *P. gingivalis*, have been observed to preferentially accumulate within vesicles. Notably, gingipains possessing a C-terminal regulatory domain (CTD) are found to be 3–5 times more abundant in *P. g*-OMVs compared to their levels in the bacterial cells themselves (Wielento et al., 2022). It was also noted that the protein content of *P. g*-OMVs varied among different strains. For instance, FimA, C, D, E, and Mfa1 were detected in OMVs secreted by *P. gingivalis* strain 33,277, which aligns with the expression of these proteins in that strain (Mantri et al., 2015; Veith et al., 2022). In contrast, these proteins were not detected in OMVs secreted by *P. gingivalis* strains W50 and W83 (Mantri et al., 2015; Veith et al., 2022). Through bioinformatics analysis, Veith et al. (2014) functionally categorized the identified proteins, revealing that they are primarily associated with the virulence, metabolism, and structural integrity of *P. gingivalis*. The substantial enrichment of virulence factors in *P. g*-OMVs suggests that these vesicles play a critical role in mediating adhesion, invasion, host cell damage, and the regulation of host immune responses, thereby providing a significant molecular basis for understanding the pathogenic mechanisms of *P. gingivalis*. The subsequent sections will primarily describe the pathogenic mechanisms of *P. g*-OMVs and their associated virulence factors (Figure 3).

### 3.1 Adhesive and invasive roles

The interaction between *P. gingivalis* and other bacteria is typically established through specific recognition mechanisms involving adhesins and receptors (Xie, 2015). Research has identified several components within *P. g*-OMVs, including gingivalin, FimA, PPAD, hemagglutinin A, and heat shock proteins, that contribute to the formation of plaque biofilms (Zhang et al., 2021; Xie, 2015; Abe et al., 2004). An earlier study conducted by Kamaguchi et al. (2003) demonstrated that *P. g*-OMVs facilitate the coaggregation of a diverse array of oral microorganisms, encompassing various species of *Streptococcus* spp., *Fusobacterium nucleatum* (*F. nucleatum*), *Actinomyces naeslundii* (*A. naeslundii*), *Actinomyces viscosus* (*A. viscosus*), Spirochaetes, and *Aggregatibacter actinomycetemcomitans* (*A. actinomycetemcomitans*). Furthermore, the presence of *P. g*-OMVs was shown to induce coaggregation between *Staphylococcus aureus* (*S. aureus*) and *Streptococcus* spp., as well as with the mycelial forms of *Candida albicans* (*C. albicans*). Notably, both Methicillin-Resistant *S. aureus* (MRSA) and Methicillin-Sensitive *S. aureus* (MSSA) exhibited similar coaggregation capabilities with *Streptococcus* spp., *A. naeslundii*, and *A. viscosus* in the presence of *P. g*-OMVs. Additionally, Espina et al. (2022) reported that *P. g*-OMVs could induce coaggregation of *S. aureus* in a manner dependent on PPAD and gingipains. The CTD family of proteins, primarily gingipains, which are selectively enriched in *P. g*-OMVs, also play a role in bacterial coaggregation, promote biofilm formation, and serve as intermediaries in the transport of non-ergotoxigenic bacteria by ergotoxigenic bacteria (Veith et al., 2022). Consequently, *P. g*-OMVs can interact with other bacterial species, thereby participating in the regulation of biofilm formation through the conveyance of their own virulence factors with adhesive properties. This indicates that *P. g*-OMVs may facilitate communication with other oral bacteria on behalf of *P. gingivalis*. The invasive capacity of *P. g*-OMVs has been well-documented, primarily attributed to their adhesion to host cells (Tribble and Lamont, 2010). FimA is a well-characterized adhesin that mediates the adhesion and invasion of *P. gingivalis* to host cells. However, it has been established that fimbriae are not essential for the invasive capability of *P. g*-OMVs; rather, a significant quantity of long fimbriae is necessary for effective invasive activity (Yilmaz et al., 2002). Conversely, the FimA protein within *P. g*-OMVs enhances the invasion of *P. gingivalis* into host tissues from periodontal ulcer sites by interacting with cell surface receptors (Rothenberger et al., 2024; Yilmaz et al., 2002). The accumulation of virulence factors on the surface of *P. g*-OMVs provides a strategic advantage by evading degradation through protein hydrolysis, thereby facilitating more efficient adhesion and invasion of host cells and enabling dissemination through the bloodstream to distant organs (Okamura et al., 2021). Evidence suggests that *P. g*-OMVs can be internalized by host cells via actin-mediated receptor pathways and gross peptide lipid raft endocytosis (Furuta et al., 2009). Additionally, *P. g*-OMVs can induce cell membrane ruffling, allowing them to persist within the lysosomes of host cells and effectively activate Toll-like receptor 2 (TLR2) (Furuta et al., 2009; Zou et al., 2024). Beyond their intrinsic adhesive and invasive properties, *P. g*-OMVs can also significantly influence the adhesion and invasion capabilities of other bacteria toward host cells. For instance, Inagaki et al. (2006) demonstrated that *P. g*-OMVs could modulate the adhesion and invasion of *Tannerella forsythia* (*T. forsythia*) on gingival epithelial cells by promoting the expression of the leucine-rich repeat sequence of the BspA protein in *T. forsythia*. More recently, Zhang et al. (2022) found that gingipains present in *P. g*-OMVs could downregulate the expression of surface adhesion-related proteins FadA and FomA in *F. nucleatum*, thereby inhibiting its invasion of gingival epithelial cells. Furthermore, Rothenberger et al. (2024) identified the specific lipoprotein PG1881 in *P. g*-OMVs, which binds to multiple receptors on the host cell surface, similar to fimbriae, thereby enhancing the adhesion of *P. gingivalis* to erythrocytes. This interaction enables *P. gingivalis* to firmly attach to erythrocyte surfaces and evade the host’s immune clearance mechanisms, thereby creating favorable conditions for its survival and proliferation within the host. Interestingly, *P. g*-OMVs also possess the ability to promote adhesion among host cells. Ho et al. (2016) reported that *P. g*-OMVs significantly enhanced the adhesion of monocytes to the monolayer of human umbilical vein endothelial cells (HUVECs). Additionally, the expression of C-X-C motif chemokine ligand 8 (CXCL8) and endothelial-leukocyte adhesion molecule 1 (E-selectin) induced by *P. gingivalis* was found to be elevated in HUVECs, correlating with the invasive capacity of *P. g*-OMVs.

### 3.2 Immunomodulatory effects

One of the functional advantages of *P. g*-OMVs is their high immunogenicity. Nakao et al. (2014) demonstrated that sera from patients with periodontitis exhibited significantly greater reactivity to wild-type strains producing *P. g*-OMVs compared to strains deficient in *P. g*-OMVs, indicating that these vesicles serve as a crucial source of antigens. Furthermore, *P. g*-OMVs display antigen specificity, as the gingipains contained within them can elicit an antibody-mediated immune response against *P. gingivalis* infection. *P. g*-OMVs also possess notable functional advantages in modulating the activation and response of immune cells. Previous research has shown that *P. g*-OMVs induce the infiltration of neutrophils into connective tissue and are involved in promoting the formation of foam cells by macrophages (Yilmaz et al., 2006; Qi et al., 2003). Additionally, Nakao et al. (2016) utilized a mouse model to demonstrate that intranasal inoculation with *P. g*-OMVs resulted in a dose-dependent increase in serum immunoglobulin G (IgG), including IgG1 and IgG2a, as well as salivary secretory IgA (S-IgA) expression, which was sustained for at least 28 weeks and 18 weeks post-immunization, respectively. In contrast, the effects of the parental bacterial strains were not significant. A recent *in vivo* study by Nakao et al. (2023) revealed that the combination of *P. g*-OMVs with *A. actinomycetemcomitans* OMVs (*A. a*-OMVs) significantly enhanced the immune response specific to *P. gingivalis*, resulting in elevated serum IgG and salivary IgA levels, as well as promoting the coaggregation of *P. gingivalis* and *A. actinomycetemcomitans*. Further investigations indicated that oral administration of both *P. gingivalis* and *A. actinomycetemcomitans* following intranasal immunization with the combination of *P. g*-OMVs and *A. a*-OMVs led to a significant reduction in the presence of both microorganisms in the oral cavity of mice. This finding suggests that intranasal immunization with *P. g*-OMVs may be effective in preventing the colonization of periodontal pathogens in the oral cavity and mitigating the development of systemic diseases associated with periodontitis. Moreover, *P. g*-OMVs have been shown to exert significant immunomodulatory effects on monocytes and macrophages. Cecil et al. (2017) found that after phagocytosis by monocytes and macrophages, *P. g*-OMVs induced the secretion of pro-inflammatory cytokines, including tumor necrosis factor-alpha (TNF-α), interleukin (IL)-8, and IL-1β, through the activation of nuclear factor kappa-light-chain-enhancer of activated B cells (NF-κB). Additionally, *P. g*-OMVs promote the formation of the NLRP3 inflammasome complex in macrophages and stimulate the secretion of pro-inflammatory cytokines both *in vitro* and in vivo via the activation of the NOD-like receptor family pyrin domain containing 3 (NLRP3) inflammasome (Cecil et al., 2017). *P. g*-OMVs function as a virulence factor secretion system that enhances pathogenic effects, in part by facilitating the evasion of host immune defense mechanisms (Duncan et al., 2004). Duncan et al. (2004) provided evidence that the recombinant human CD14 receptor is susceptible to hydrolytic degradation by proteins derived from *P. g*-OMVs. This study indicates that *P. g*-OMVs lead to the loss of membrane-bound CD14 receptors and that gingipains degrade receptors for LPS, resulting in a diminished macrophage response to LPS stimulation. This may enhance the ability of *P. gingivalis* and other periodontal pathogens to evade host immune responses. Furthermore, gingipains present on *P. g*-OMVs degrade and inactivate various immunoglobulins and cytokines, thereby protecting *P. gingivalis* from phagocytosis and facilitating its entry into the bloodstream, which can lead to bacteremia (Wielento et al., 2022; Bregaint et al., 2022). Wielento et al. (2022) concluded that PPAD is an essential component for *P. g*-OMVs to activate TLR2 and subsequently induce pro-inflammatory cytokine production by host cells. This is primarily attributed to the ability of PPAD to enhance TLR2 activation by fimbriae through citrullination modification. Thus, *P. g*-OMVs play significant roles in inducing immune responses and regulating immune cell functions, as well as evading immune defenses through various mechanisms, thereby contributing to the complex dual role of *P. g*-OMVs in the pathogenesis of periodontitis and related systemic diseases.

### 3.3 Damage to host cells

Following the invasion of host cells, *P. g*-OMVs can inflict damage through various mechanisms. LPS, a principal component of the bacterial outer membrane, plays a significant role in stimulating the innate immune response and is a critical virulence factor that triggers cytokine production (Rothenberger et al., 2024; Koeppen et al., 2016). Research has demonstrated that LPS derived from *P. g*-OMVs can enhance the secretion of pro-inflammatory cytokines from gingival epithelial cells by engaging the TLR/NF-κB signaling pathway (Kou et al., 2008). Furthermore, *P. g*-OMVs disrupt the normal metabolic and physiological functions of host cells by degrading biologically active substances, thereby inhibiting cellular proliferation and impairing the physiological functions of epithelial cells. For instance, Furuta et al. (2009) reported that the gingipains present in *P. g*-OMVs could deplete transferrin levels in host cells and inhibit cell migration by degrading the transferrin receptor and proteins associated with adhesion junction complexes. Recent studies have also indicated that *P. g*-OMVs are implicated in the programmed death of host cells. Fan et al. (2023) found that the uptake of *P. g*-OMVs by human periodontal ligament cells resulted in apoptosis, which subsequently promoted the release of pro-inflammatory cytokines, including IL-6 and IL-8, thereby inducing cellular dysfunction. Additionally, Fleetwood et al. (2017) observed that *P. g*-OMVs induced pyroptosis in macrophages. Moreover, Huang et al. (2023) discovered that *P. g*-OMVs accelerated endothelial dysfunction by promoting mitochondria-associated cell death in human retinal microvascular endothelial cells (HRMECs).

The pathogenic effects exerted by the virulence factors encapsulated within *P. g*-OMVs are intrinsically linked to their distinctive protective structure. *P. g*-OMVs are composed of bilayer lipid membranes that are enriched with LPS and OMPs, such as OmpA. This configuration forms a physical barrier that safeguards the internal cargo from degradation by host proteases, antimicrobial peptides, and oxidative stress, while preserving the functionality of the virulence factors. *In vivo* experiments conducted by Seyama et al. (2020) demonstrated that *P. g*-OMVs, when injected via the tail vein, are capable of reaching the liver through the circulatory system. Upon arrival, gingipains are released, which ultimately disrupt the host’s insulin signaling pathway. This observation suggests that *P. g*-OMVs possess protective mechanisms that prevent degradation by host proteases during transit, thereby ensuring the virulence factor can exert its pathogenic effects. Furthermore, the genetic material contained within *P. g*-OMVs exhibits a certain degree of stability against host nucleic acid-degrading enzymes; however, the specific mechanisms that confer this resistance require further investigation. Martinez-Martinez et al. (2009) identified the presence of *P. gingivalis* DNA in patients diagnosed with rheumatoid arthritis (RA), although *P. gingivalis* itself was not detected in the joint fluid. This observation suggests that *P. g*-OMVs may serve as significant vectors for the transport of *P. gingivalis* DNA to distant organs, circumventing the necessity for direct translocation of the bacterium. The transfer of genetic material encapsulated within *P. g*-OMVs to host cells and remote target organs has the potential to elicit pathogenic effects. Koeppen et al. (2016) found that mRNAs within *P. g*-OMVs can be transcribed and translated upon entering host cells, while sRNAs, which contain various packages, have the potential to influence host mRNA function and stability. Additionally, sRNAs can mediate host immune responses by regulating host gene expression (Diallo and Provost, 2020; Liu et al., 2021). Moreover, *P. g*-OMVs possess the capability to activate multiple intracellular signaling pathways and serve as a communication mechanism between bacteria or between bacteria and host cells (Johnston et al., 2023; Zhang et al., 2021). These findings indicate that the pathogenic role of *P. g*-OMVs is significant not only in the progression of periodontitis but may also play a critical role in the development of periodontitis-associated systemic diseases. Consequently, *P. g*-OMVs are essential for both oral and systemic health.

## 4 Pathogenic role and mechanism of *P. g*-OMVs in periodontitis

The growth process of *P. gingivalis* results in the production of numerous virulence factors that elicit a host immune response. This response can independently or synergistically mediate inflammation and injury to periodontal tissues, ultimately leading to tooth loss (Takeuchi et al., 2019; Amano et al., 2014). The interaction between plaque microorganisms and the host’s immune-inflammatory response is a critical determinant of the extent of periodontal tissue destruction. Periodontitis, classified as a chronic infectious inflammatory disease, is initiated by the presence of dental plaque biofilm (Yoshimura et al., 2009; Ho et al., 2015). It has been previously noted that the virulence factors of *P. g*-OMVs, which possess adhesive properties, are capable of inducing coaggregation among a diverse array of oral microorganisms. This coaggregation contributes to the formation of plaque biofilms. The oral microorganisms and their metabolites that aggregate within the plaque stimulate the host to mount an immune-inflammatory response, which subsequently leads to the progressive destruction of periodontal tissues.

Previous research has demonstrated that *P. g*-OMVs, similar to *P. gingivalis*, possess the ability to invade or stimulate host cells, including gingival epithelial cells, gingival fibroblasts, and periodontal membrane cells. This interaction subsequently elicits a range of responses in host cells, such as inflammation, programmed cell death, and immune responses (Furuta et al., 2009; Martinez-Martinez et al., 2009). Previous research has examined the invasion efficiency of *P. g*-OMVs in comparison to that of *P. gingivalis* itself. The findings indicated that 70–90% of primary human gingival epithelial cells and gingival fibroblasts internalized *P. g*-OMVs after 1 h of exposure, whereas only 20–50% of the host cells internalized *P. gingivalis*. These results suggest that *P. g*-OMVs exhibit a significantly higher efficiency of invasion and internalization compared to their parent bacterium, *P. gingivalis* (Ho et al., 2015). Furthermore, Kou et al. (2008) observed that immortalized human gingival epithelial cells co-cultured with *P. g*-OMVs exhibited elevated expression levels of inflammation-related factors, including cyclooxygenase (COX)-2, IL-6, IL-8, MMP-1, and MMP-3. IL-6 and IL-8 are critical pro-inflammatory cytokines implicated in the pathogenesis of periodontitis. Several studies have elucidated the potential mechanisms by which *P. g*-OMVs enhance IL-8 and IL-6 expression in human gingival epithelial cells. Uemura et al. (2022) reported that *P. g*-OMVs induced an increase in IL-8 and IL-6 expression in human gingival epithelial cells through the activation of extracellular signal-regulated kinase (Erk)1/2, c-Jun N-terminal kinase (JNK), p38 mitogen-activated protein kinase (MAPK), and NF-κB following co-culture with *P. g*-OMVs. Additionally, Uemura et al. (2022) demonstrated that *P. g*-OMVs also stimulated increased expression of IL-6 and IL-8 in human gingival epithelial cells via the activation of the stimulator of interferon genes (STING), a pivotal innate immune signaling molecule that recognizes cellular or microbial DNA in the cytoplasm. This finding suggests that the DNA present in *P. g*-OMVs may play a role in promoting the onset and progression of periodontitis. Chen et al. (2024) demonstrated that *in vitro*, *P. g*-OMVs, upon endocytosis by ECs, directly induced endothelial dysfunction. This process activated the cGAS-STING-TBK1 signaling pathway within ECs, which subsequently inhibited the migration of MG63 cells (a human osteosarcoma cell line), impeded their early osteogenic differentiation, and disrupted the normal osteogenic process. *In vivo*, *P. g*-OMVs were found to promote alveolar bone resorption, elevate vascular STING levels, reduce the number of Runx2+ cells surrounding the alveolar bone, and impair endothelial cell-mediated osteogenic responses. The promotion of osteoclast differentiation, along with the damage to periodontal cells, contributes significantly to the role of *P. g*-OMVs in the pathogenic mechanisms underlying periodontitis. Zou et al. (2024) reported that *P. g*-OMVs could facilitate osteoclast differentiation by delivering LPS to activate TLR2. Furthermore, Fan et al. (2023) elucidated that *P. g*-OMVs were implicated in the pathogenesis of periodontitis both *in vivo* and in vitro. An *in vitro* investigation revealed substantial alveolar bone resorption in rats stimulated with *P. g*-OMVs. Additionally, in vitro studies indicated that following the uptake of *P. g*-OMVs by human periodontal cells, microRNA-sized small RNA (msRNA) sRNA45033 present in *P. g*-OMVs regulated the methylation and expression of p53 DNA by targeting chromobox 5 (CBX5), which ultimately resulted in apoptosis and the release of pro-inflammatory cytokines.

*Porphyromonas gingivalis* OMVs exert pathogenic effects by interacting with periodontal immune cells. For instance, Cecil et al. (2016) demonstrated that *P. g*-OMVs enhance the production of TNF-α, IL-1β, and IL-6 through the activation of pattern recognition receptors (PRRs) on macrophages and monocytes within periodontal tissues. These pro-inflammatory cytokines contribute to the destruction of connective tissue and the resorption of alveolar bone, which are characteristic clinical features of periodontitis (Cecil et al., 2017; Kwon et al., 2021). Furthermore, Fleetwood et al. (2017) stimulated gingival macrophages with *P. gingivalis* and *P. g*-OMVs, respectively, and observed that macrophages in the *P. gingivalis*-infected group did not exhibit significant activation of the NLRP3 inflammasome or characteristics of pyroptotic cell death. In contrast, the NLRP3 inflammasome in the *P. g*-OMVs group was significantly activated, leading to a marked upregulation of cysteine aspartate protease-1 (caspase-1) and the substantial release of cellular contents such as IL-1β, IL-18, and lactate dehydrogenase (LDH). The observed cellular features included nuclear contraction, chromatin disruption, and membrane swelling and rupture, indicating the occurrence of pyroptosis. These findings suggest that *P. g*-OMVs exert a more potent pyrogenic effect on gingival macrophages compared to *P. gingivalis*.

**Figure 4:**
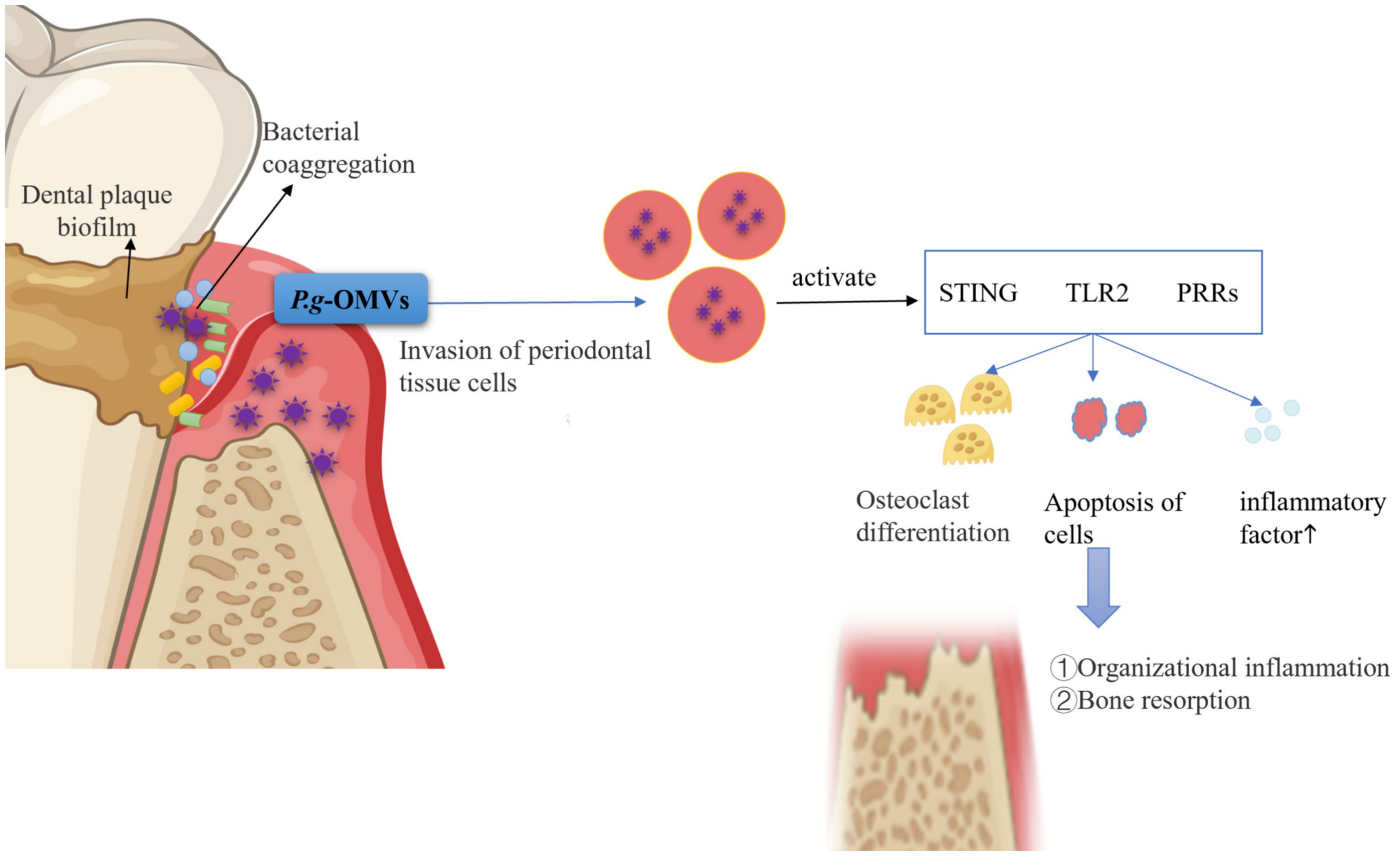
Pathogenic role of *P. g*-OMVs and associated mechanisms in periodontitis. *P. g*-OMVs facilitate the aggregation of various *Streptococcus* spp., *F. nucleatum*, Spirochaetes, *A. actinomycetemcomitans*, and other bacteria within plaque biofilms, thereby mediating interactions with other oral microorganisms on behalf of *P. gingivalis*. Furthermore, *P. g*-OMVs possess the capability to invade or stimulate periodontal cells, leading to the activation of receptors such as STING, TLR2, and PRRs. This activation subsequently induces osteoclast differentiation, inflicts damage on periodontal cells, and promotes the release of pro-inflammatory cytokines, ultimately resulting in alveolar bone resorption and a cellular immune-inflammatory response within periodontal tissues. STING, stimulator of interferon genes; TLR2, toll-like receptor 2; PRRs, pattern recognition receptors.

In summary, *P. g*-OMVs play a significant role in the establishment of an inflammatory microenvironment within periodontal tissues, which can lead to subsequent alveolar bone destruction. This occurs through the mediation of oral microbial coaggregation, invasion, and the stimulation of host cells (Figure 4). This indicates that *P. g*-OMVs may play a crucial role as a pathogenic component of *P. gingivalis* in the stimulation of host cells, the modulation of local immune and inflammatory responses, and the induction of the onset and progression of periodontitis.

## 5 Pathogenic role of *P. g*-OMVs in systemic diseases and mechanisms

**Figure 5:**
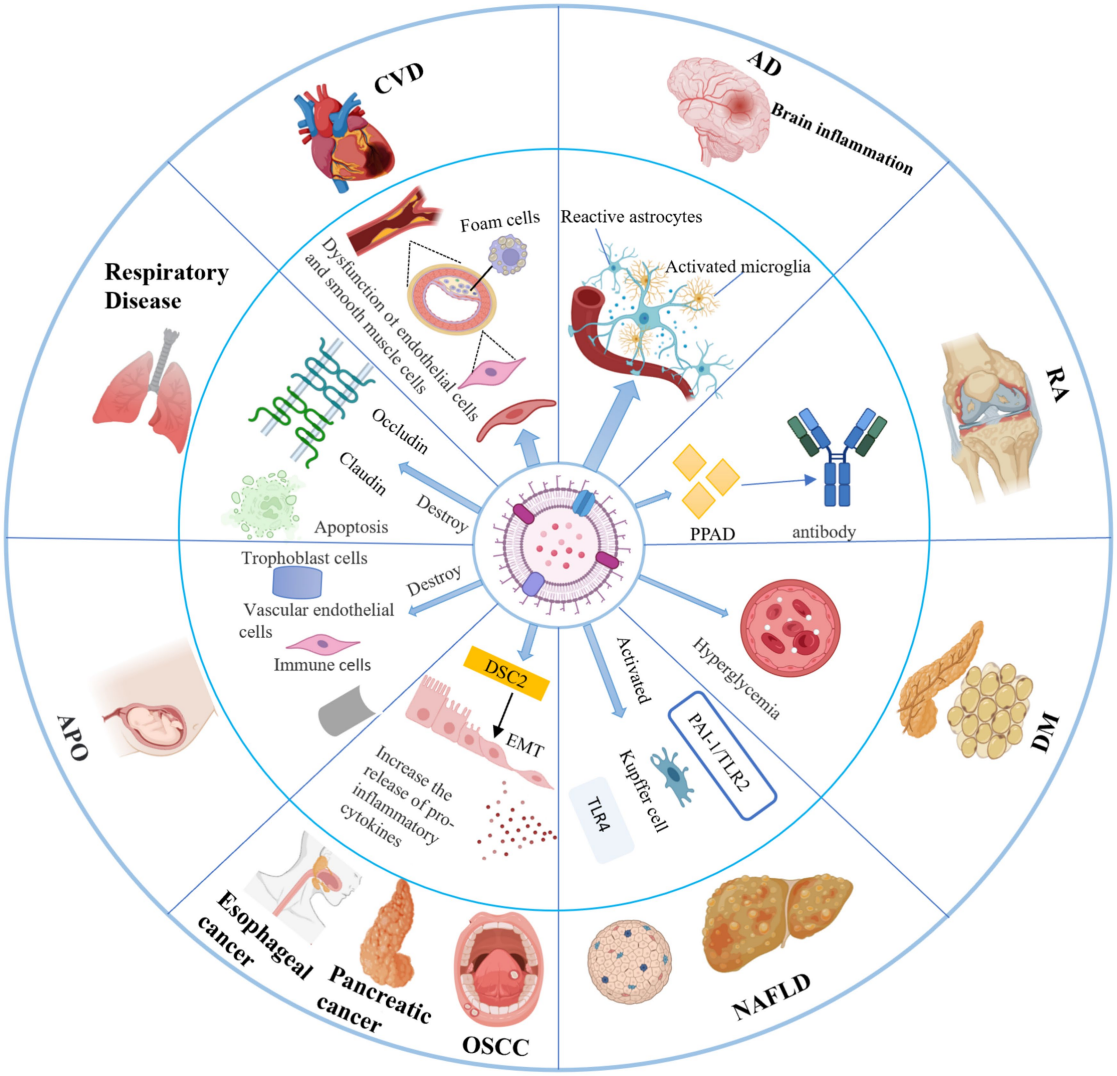
Pathogenic role of *P. g*-OMVs in systemic diseases and related mechanisms. *P. g*-OMVs are closely associated with the development of a variety of systemic diseases, including cardiovascular disease, Alzheimer’s disease, rheumatoid arthritis, diabetes mellitus, nonalcoholic fatty liver disease, cancers, adverse pregnancy outcomes, and respiratory diseases. CVD, cardiovascular disease; AD, Alzheimer’s disease; RA, rheumatoid arthritis; DM, diabetes mellitus; NAFLD, non-alcoholic fatty liver disease; APO, adverse pregnancy outcome; OSCC, oral squamous cell carcinoma; EMT, epithelial-mesenchymal transition; DSC2, desmoglein-2; PPAD, peptidylarginine deiminase.

Compared to parental cells, *P. g*-OMVs are distinguished by their diminutive size and high abundance, as well as their well-developed membrane structure, stability, and resistance to proteases derived from the host. These characteristics facilitate the effective invasion of host tissues by *P. g*-OMVs and enable their diffusion through the bloodstream to distant organs and regions that are inaccessible to the parental bacteria. As a result, the pathogenic effects of *P. gingivalis* are amplified not only within the oral cavity but also in various other areas of the body (Kadurugamuwa and Beveridge, 1995; Okamura et al., 2021). Nonaka et al. (2024) demonstrated that *P. g*-OMVs can internalize *P. gingivalis* into human intestinal cells, subsequently translocating into the cytoplasm, where they directly degrade occludin on the cytoplasmic side. This degradation leads to increased permeability, thereby inducing dysfunction of the intestinal barrier. The intestinal barrier is a critical component of the immune system’s defense, and its proper functioning is essential for maintaining immune homeostasis. Disruption of the intestinal barrier can precipitate immune abnormalities, which are closely linked to the onset and progression of systemic diseases through various mechanisms (Figure 5). These findings suggest that the pathogenic effects of *P. g*-OMVs may provide a fundamental basis and a key mechanism by which *P. gingivalis*-induced periodontal infections contribute to the occurrence and development of systemic diseases.

### 5.1 Cardiovascular diseases

Cardiovascular disease (CVD) is recognized as the leading cause of mortality worldwide. A substantial body of evidence from epidemiological, clinical, and experimental studies indicates a significant association between periodontitis and the development of CVD, suggesting that periodontitis may function as an independent risk factor for this condition (Farrugia et al., 2020; Guo et al., 2024). *P. gingivalis*, a key pathogenic bacterium associated with periodontitis, has been the subject of extensive research, revealing that both *P. gingivalis* and its virulence factors significantly contribute to the pathogenesis of atherosclerosis (As) and may be central to the relationship between periodontitis and CVD (Kozarov et al., 2005; Ye et al., 2022; Yang et al., 2016). Although biomolecules, such as nucleic acids from *P. gingivalis*, are frequently detected in cardiovascular samples, establishing the presence of viable bacteria remains a challenge. It has been demonstrated that previously identified biomolecules, including nucleic acids from *P. gingivalis*, are likely derived from *P. g*-OMVs (Kozarov et al., 2005). Furthermore, several clinical studies have indicated that systemic antibiotic therapy does not effectively reduce vessel wall inflammation or the subsequent occurrence of adverse cardiovascular events and mortality in patients with CVD (Gui et al., 2016; Zhang et al., 2021). This finding raises questions regarding the hypothesis that *P. gingivalis* influences the development of CVD and its association with periodontitis. *P. g*-OMVs are suspected to play a pivotal role in distal cardiovascular injury due to their capacity to transport active and concentrated virulence factors to distant sites, as well as their ability to target specific locations, thereby implicating them in distal cardiovascular damage (Guo et al., 2024). Farrugia et al. (2020) demonstrated that *P. g*-OMVs containing gingipains increase the permeability of cardiovascular cells. Additionally, Guo et al. (2024) found that *P. g*-OMVs induced pericardial enlargement, vascular damage, elevated neutrophil counts, and the activation of inflammatory pathways in zebrafish larvae. Collectively, these studies provide preliminary insights into the potential mechanisms by which *P. g*-OMVs may be involved in the pathogenesis of CVD. Nevertheless, the fundamental aspects concerning the cytological basis and molecular mechanisms by which *P. g*-OMVs facilitate the progression of CVD remain inadequately understood.

As a recognized pathological foundation for numerous CVDs, this condition is characterized as a chronic inflammatory disorder that primarily affects large and medium-sized arteries. The disease is influenced by various factors that elicit an inflammatory response, ultimately resulting in dysfunction of vascular endothelial cells, impairment of smooth muscle cells, and a cascade of inflammation-associated pathological processes (Farrugia et al., 2020; Xie et al., 2020; Jia et al., 2015). Research has demonstrated that LPS derived from *P. gingivalis* enhance the secretion of pro-inflammatory cytokines, including IL-1β, IL-6, and TNF-*α*, via the TLR/NF-κB signaling pathway. This activation subsequently induces significant oxidative stress in ECs (Xie et al., 2020). Furthermore, the potential role of LPS derived from *P. g*-OMVs in modulating the TLR/NF-κB signaling pathway to facilitate the progression of As warrants further investigation. Calcium deposition within the vessel wall is a hallmark of advanced As. A study conducted by Yang et al. (2016) revealed that *P. g*-OMVs can induce calcification in vascular smooth muscle cells by activating the extracellular signal-regulated kinase 1/2 (ERK1/2)-runt-related transcription factor 2 (RUNX2) pathway. Additionally, platelet aggregation is considered a critical factor in the formation of As plaques, and *P. g*-OMVs have the capacity to enter the bloodstream from the site of infection, thereby exhibiting significant platelet aggregation activity (Sharma et al., 2000). Consequently, *P. g*-OMVs may play a role in the development of As and thromboembolic events. The formation of foam cells is a defining event in the progression of As. It has been reported that *P. g*-OMVs released locally from periodontitis lesions into the circulation can transfer virulence factors to the arterial wall, stimulate foam cell formation in murine macrophages, and promote the coaggregation of low-density lipoprotein cholesterol (LDL), thereby facilitating the advancement of As (Qi et al., 2003; Miyakawa et al., 2004). Nitric oxide (NO) plays a crucial role in maintaining the normal function of the cardiovascular system by regulating vasodilation, inhibiting platelet aggregation, and mitigating inflammatory responses. Endothelial nitric oxide synthase (eNOS) is a pivotal enzyme that catalyzes the conversion of l-arginine to NO. However, the inhibition of eNOS expression results in decreased NO production or diminished bioactivity, which contributes to endothelial dysfunction and exacerbates the progression of As. Research has demonstrated that *P. g*-OMVs can inhibit eNOS expression through the activation of Rho-associated kinase (ROCK) in HUVECs via the ERK1/2 and p38 MAPK signaling pathways, leading to vascular endothelial dysfunction (Jia et al., 2015). Furthermore, one study indicated that *P. g*-OMVs, through ROCK activation, can induce the formation of stress fibers and the degradation of vascular endothelial adhesion proteins (VEc) via lysosomal and endoplasmic reticulum-mediated degradation in HUVECs, thereby increasing the permeability of vascular endothelial cells (Mekata et al., 2024).

The studies referenced indicate that *P. g*-OMVs may significantly contribute to the pathogenicity of *P. gingivalis* in the onset and progression of CVD. Additionally, there is an increasing body of evidence suggesting that the mitochondrial autophagy/dysfunction-NLRP3 inflammasome-cellular pyroptosis pathway is involved in the development of CVD, including As. *P. g*-OMVs have been demonstrated to induce cellular inflammation, increase levels of reactive oxygen species (ROS), provoke mitochondrial dysfunction, activate the NLRP3 inflammasome, and initiate both cell death and endothelial dysfunction. These processes may substantially affect the onset and progression of microvascular diseases. Endothelial dysfunction is recognized as a critical factor in the pathogenesis of As. Therefore, it is hypothesized that *P. g*-OMVs may contribute to the development and progression of As by inducing endothelial dysfunction through the modulation of the mitochondrial dysfunction-NLRP3 inflammasome-cellular pyroptosis pathway. However, this hypothesis necessitates validation through further research. Investigating the mechanisms by which *P. g*-OMVs contribute to the occurrence and progression of CVD not only enhances the understanding of the pathogenic mechanisms associated with *P. g*-OMVs but may also provide new insights into the relationship between periodontitis and CVD.

### 5.2 Alzheimer’s disease

Alzheimer’s disease (AD) is recognized as the most prevalent chronic neurodegenerative disorder, characterized by clinical manifestations such as impairments in learning and memory, as well as cognitive dysfunction (Singhrao and Olsen, 2018). The pathological features of AD include the abnormal accumulation of *β*-amyloid (Aβ), hyperphosphorylation of tau protein, neuroinflammatory responses, and neuronal loss within the brain (Leng and Edison, 2021; Villemagne et al., 2013). The etiology of AD and its underlying pathophysiological mechanisms remain inadequately understood, particularly with respect to the role of periodontal pathogenic bacteria. Current hypotheses propose that bacteria associated with periodontitis may contribute to the onset of AD through two primary mechanisms. The first mechanism involves an indirect effect on the central nervous system via disruption of the microbiota-gut-brain axis, which leads to chronic inflammation in the brain and subsequent neuroinflammatory responses. The second mechanism is attributed to compromised local barrier function, resulting in ulcerative defects in the inner walls of periodontal pockets. This condition increases the likelihood that periodontal pathogenic bacteria, along with their virulence factors and metabolites, can traverse neural pathways or enter the bloodstream, ultimately reaching brain tissue and exerting pathogenic effects across the blood–brain barrier (BBB) (Dominy et al., 2019; Liu et al., 2024).

*Porphyromonas gingivalis*, a predominant pathogen within the subgingival microbial community associated with chronic periodontitis, is currently recognized as the most extensively studied periodontal pathogen with potential neuropathological implications for AD (Singhrao and Olsen, 2018). Previous research has suggested that *P. gingivalis* and its byproducts may influence the pathogenesis of AD; however, viable *P. gingivalis* has not been isolated from human brain tissue, and imaging studies have not successfully identified clusters of *P. gingivalis* cells within brain tissue (Yoshida et al., 2023; Liu et al., 2024). Furthermore, various components of *P. gingivalis*, including nucleic acids, LPS, and gingipains, have been detected in multiple regions of the human brain affected by AD, such as the cortical gray matter, basal forebrain, and hypothalamic areas. Notably, these components can be transported by *P. g*-OMVs (Dominy et al., 2019; Liu et al., 2024). This observation raises the question of whether *P. g*-OMVs, as carriers of diverse bacterial biomolecules, serve as a mechanistic link between *P. gingivalis* and the pathophysiology of AD in the brain. A study conducted by Gong et al. (2022) demonstrated that *P. g*-OMVs localize to the hippocampus and cortex, contributing to memory dysfunction, neuroinflammation, and tau protein phosphorylation in middle-aged mice, while also activating NLRP3 inflammatory vesicles. Additionally, this study identified the presence of *P. g*-OMVs in the trigeminal ganglion and proposed that these vesicles may translocate to the brain via the trigeminal nerve, utilizing nerve endings located in periodontal tissue (Gong et al., 2022).

The BBB is a highly selective and permeable structural barrier that separates peripheral blood circulation from the central nervous system. Under normal physiological conditions, the BBB allows the passage of only hydrophobic molecules with molecular weights less than 400 Da, serving as the primary defense mechanism of the brain against the invasion of pathogenic microorganisms (Carter, 2017). Dominy et al. (2019) were the first to demonstrate the presence of DNA and gingipains from *P. gingivalis* in the brain tissue of patients with AD through molecular assays, including quantitative polymerase chain reaction (qPCR) and immunohistochemistry. They specifically noted the presence of the gingipains RgpB and Kgp, which have been shown to induce abnormal coaggregation and hyperphosphorylation of tau proteins by directly cleaving them. This finding suggests that the virulence factors of *P. gingivalis* can penetrate the brain via the BBB and exert pathogenic effects. Recent studies have indicated that circulating *P. g*-OMVs, upon uptake by brain ECs, can disrupt cellular and subcellular structures and functions, ultimately leading to cell death and increased BBB permeability (Nonaka et al., 2022). *In vitro* experiments have demonstrated that *P. g*-OMVs carry gingipains that are internalized by human brain microvascular ECs, resulting in the degradation of intracellular tight junction proteins, specifically ZO-1 and occludin (Nonaka et al., 2022). This degradation disrupts intercellular tight junctions and contributes to an increase in BBB permeability. Similarly, Pritchard et al. (2022) showed that *P. g*-OMVs, which also carry gingipains into human brain microvascular ECs, can degrade the tight junction protein ZO-1 and, akin to *P. gingivalis*-derived LPS, can compromise the integrity of BBB models and reduce BBB resistance (Pritchard et al., 2022). The increase in BBB permeability is closely associated with a decrease in BBB resistance, and the integrity of the BBB is essential for maintaining homeostasis and normal neurological function in the brain. Disruption of BBB integrity and function can lead to the accumulation of harmful substances, such as inflammatory factors, Aβ, and neurotoxic agents, which may exacerbate the pathological processes of AD by triggering neuroinflammation, accelerating Aβ deposition, and affecting tau protein phosphorylation (Liu et al., 2024).

Microglia serve as resident immune cells within the central nervous system, and their activation-induced inflammatory response constitutes a significant contributor to neuroinflammation in AD (Leng and Edison, 2021). Neuroinflammation has the potential to exacerbate neuronal cell damage by facilitating the deposition of Aβ and the phosphorylation of tau protein in brain tissue, ultimately leading to cognitive decline (Villemagne et al., 2013). Yoshida et al. (2023) demonstrated that repeated intraperitoneal injections of *P. g*-OMVs in mice over a 12-week period resulted in the translocation of these vesicles into the ventricles of the mouse brain. Notably, *P. g*-OMVs that reached the ventricles were found to enhance the Tau/Tau phosphorylation ratio in the brain, activate microglia, and induce the expression of pro-inflammatory cytokines in the human microglial cell line MHC3 in a manner dependent on gingipains. Furthermore, Inoue et al. (2024) reported that the uptake of *P. g*-OMVs by microglial cells stimulated the production of IL-1β through the activation of the NF-κB signaling pathway. Additionally, a study conducted by Chuang et al. (2024) revealed that microglia could internalize *P. g*-OMVs, which subsequently promoted the production of pro-inflammatory cytokines. This mechanism was primarily mediated by the LPS component of *P. g*-OMVs through the activation of the phosphorylated AKT and phosphorylated JNK signaling pathways. The investigation conducted by Chuang et al. (2024) revealed that gingipains play a mediating role in the neurotoxicity induced by *P. g*-OMVs in SH-SY5Y neuroblastoma cells. These findings indicate that *P. g*-OMVs, along with their virulence factors, can penetrate the ventricles, leading to neuroinflammation and neurotoxicity through the activation of host cells. Cathepsin B (CatB), a lysosomal cysteine protease, is known to have a significant association with AD when its expression is aberrant or its activity is dysregulated. CatB contributes to the hallmark pathological features of AD by activating the stress-activated protein kinase (SAPK)/Jun amino-terminal kinase (JNK) signaling pathway, which exacerbates the neuroinflammatory response and promotes the deposition of Aβ and the phosphorylation of tau protein (Wu et al., 2017). Jiang et al. (2024) demonstrated that exposure to *P. g*-OMVs resulted in increased activity and expression of CatB, which, through the regulation of the SAPK/JNK pathway, induced microglia-mediated neuroinflammation, neuronal tau phosphorylation, and synaptic loss, ultimately leading to cognitive dysfunction in mice. The pathological alterations observed in AD encompass a variety of cell types, including neurons, astrocytes, vascular endothelial cells, pericytes, and oligodendrocytes, in addition to microglia. The pathological changes in these cell types interact with one another and collectively contribute to the progression of AD. Consequently, future research should investigate the pathogenic effects of *P. g*-OMVs on other cell types involved in the pathological changes associated with AD to further elucidate the underlying mechanisms of *P. g*-OMVs in the context of this disease (Liu et al., 2024).

Iron has long been implicated in the pathogenesis of AD. It has been proposed that gingipains on the surface of *P. g*-OMVs may be involved in a novel mechanism of degrading host iron-containing proteins to promote AD deterioration (Liu et al., 2024; Lane et al., 2023). It has been shown that *P. g*-OMVs load large amounts of heme and iron due to their hemolytic capacity, proteolytic degradation of hemoglobin, and other iron-carrying plasma proteins, including transferrin (Dashper et al., 2017). Therefore, *P. g*-OMVs can divert iron from the bloodstream and shift the distribution of iron from the blood to the brain through the entry of *P. g*-OMVs into the brain tissue, resulting in “iron overload” of the brain tissue. In this way, *P. g*-OMVs may become a new mechanistic link between periodontitis and AD. In addition, *P. gingivalis* on *P. g*-OMVs hydrolyzes ferritin and other iron-containing proteins and also releases iron to expand the free iron pool and accumulate iron death stress (Liu et al., 2024). Based on these findings, it has been suggested that *P. g*-OMVs are the link between the “infectious hypothesis” and the “metal hypothesis” by associating *P. g*-OMVs with the “rusty” brain (Liu et al., 2024; Liu et al., 2023).

### 5.3 Rheumatoid arthritis

Rheumatoid arthritis is an autoimmune disorder characterized by chronic, erosive polyarthritis, which may result in the destruction of articular cartilage and the joint capsule (Larsen et al., 2020; KriauciunAs et al., 2019). Recent studies have identified *P. gingivalis* infection as a potential etiological factor involved in the pathogenic mechanisms of RA. The etiology of RA has been suggested to involve an autoimmune response that is triggered by a loss of tolerance to citrullinated host proteins (Laugisch et al., 2016; Gabarrini et al., 2018). Furthermore, enzymes capable of citrullinating host proteins have been identified in *P. gingivalis* (Laugisch et al., 2016).

It has been established that the PPAD enzyme of *P. gingivalis* is implicated in the citrullination of both bacterial and host cell proteins. This process prompts the immune system to generate citrullinated antibodies against self-antigens, ultimately resulting in a loss of tolerance to citrullinated proteins in patients with RA (Laugisch et al., 2016). Furthermore, PPAD can be retained within *P. g*-OMVs through modification by A-LPS, which protects the enzyme from proteolytic degradation (Gabarrini et al., 2018). A study has identified 78 citrullinated proteins present in the OMVs of the *P. gingivalis* W83 wild-type strain (Larsen et al., 2020). Additional research has corroborated that PPAD disrupts the equilibrium of amino acids and immune complexes, thereby compromising the integrity of the human immune system and promoting the production of citrullinated antibodies (KriauciunAs et al., 2019). These findings suggest a correlation between *P. g*-OMVs and the onset and progression of RA, with the underlying mechanism linked to the loss of immune tolerance to citrullinated host proteins mediated by PPAD in *P. g*-OMVs. Moreover, it has been reported that *S. aureus*-induced bacteremia constitutes a risk factor for RA. However, Espina et al. (2022) demonstrated that *P. g*-OMVs can facilitate the coaggregation of *S. aureus* in a manner dependent on PPAD and gingipains. This indicates that PPAD within *P. g*-OMVs may also play an indirect role in the onset and progression of RA by modulating the activity of other bacterial species. In conclusion, elucidating the role and mechanisms of *P. g*-OMVs in the pathogenesis of RA is crucial for the advancement of novel therapeutic interventions and preventive strategies.

### 5.4 Diabetes mellitus

Numerous studies have demonstrated a close bidirectional relationship between the development of diabetes mellitus (DM) and periodontitis (Polak and Shapira, 2018). However, a consistent conclusion regarding the association between these two conditions has yet to be established. Previous research indicates that the severity of periodontitis significantly influences glycemic control and the development of complications in patients with DM (Ohtsu et al., 2019).

Currently, a substantial body of research has established a link between the periodontal pathogen *P. gingivalis* and the development of DM. For instance, Ohtsu et al. (2019) demonstrated that the oral administration of *P. gingivalis* resulted in mild insulin resistance in murine models, as well as an elevation in fasting blood glucose levels in streptozotocin-induced diabetic mice. More recently, *P. g*-OMVs have also been implicated in the pathogenesis of DM and its associated complications (Huang et al., 2023; Seyama et al., 2020). Seyama et al. (2020) illustrated that *P. g*-OMVs facilitate the transport of active *P. gingivalis* to the liver, where they inhibit the Akt/glycogen synthase kinase-3β (GSK-3β) signaling pathway in HepG2 hepatocytes. This inhibition subsequently reduces hepatic glycogen synthesis, thereby maintaining elevated blood glucose levels and promoting the progression of DM in mice. Chronic complications, including vascular disease, diabetes-related nephropathy, diabetes-related peripheral neuropathy, and diabetic retinopathy (DR), are prevalent among DM patients who exhibit poor long-term glycemic control (Gabarrini et al., 2018). DR is recognized as one of the leading causes of blindness and represents a significant complication of DM. The inflammatory response and mitochondrial damage are believed to play critical roles in the development of DR (Huang et al., 2023). Huang et al. (2023) found that *P. g*-OMVs induce an inflammatory response in HRMECs and promote mitochondria-associated cell death, which accelerates endothelial dysfunction and exacerbates the progression of DR.

### 5.5 Non-alcoholic fatty liver disease

Non-alcoholic fatty liver disease (NAFLD) is characterized by diffuse hepatic steatosis and is rapidly emerging as the most prevalent chronic liver disease. The incidence of NAFLD continues to rise annually; however, its pathogenesis remains incompletely understood (KriauciunAs et al., 2019; Polak and Shapira, 2018). Several studies suggest that bacterial OMVs may play a crucial role in the pathogenic mechanisms underlying NAFLD. This is attributed to the ability of OMVs to serve as carriers for the transmission of virulence factors into hepatocytes, thereby promoting hepatic inflammation, steatosis, and fibrosis (KriauciunAs et al., 2019; Polak and Shapira, 2018). It has been demonstrated that intragastric injection of fecal-derived OMVs into the liver increases pro-inflammatory cytokine and chemokine expression in hepatic sinusoidal endothelial cells (HSECs) by acting on TLR4 and activates the production of pro-fibrotic and pro-inflammatory proteins in hepatic stellate cells (Fizanne et al., 2023). OMVs of *H. pylori* increase the levels of hepatic fibrotic markers in hepatocytes, and in cells treated with OMVs, derived exosomes activate hepatic stellate cells and induce hepatic fibrosis (Zahmatkesh et al., 2022). In addition, the accumulation of bacterial DNA may be associated with the development of NAFLD; for example, translocation of OMVs of intestinal bacterial origin promotes the accumulation of bacterial DNA in hematopoietic stem cells and hepatocytes and induces hepatocellular inflammation and stellate cellular fibrosis by activating the cGAS/STING axis (Luo et al., 2022).

The infection caused by *P. gingivalis* has been identified as a potential risk factor for non-alcoholic fatty liver disease (NAFLD) (Forrester et al., 2020; Kim et al., 2024). The surface components of *P. gingivalis*, including fimbriae and LPS, are believed to contribute to the pathogenesis of NAFLD through various mechanisms. These mechanisms encompass the promotion of endotoxemia, the elicitation of inflammatory responses, the induction of oxidative stress, metabolic remodeling, macrophage polarization, and alterations in the gut microbiota (Kim et al., 2024; Gao et al., 2023). Conversely, *P. g*-OMVs contain key components and virulence factors of the parent bacteria. Consequently, *P. g*-OMVs may significantly influence the progression of NAFLD associated with the parent bacteria. Kim et al. (2024) illustrated that *P. g*-OMVs enhance liver steatosis in mice on a low-fat diet by modulating the plasminogen activator inhibitor-1 (PAI-1)/TLR2 axis. The above studies suggest new molecular mechanisms to explain the relationship between periodontitis and NAFLD.

### 5.6 Cancer

Cancer represents a significant global health challenge characterized by a multifaceted etiology, with approximately 15% of cases linked to infections caused by specific pathogenic microorganisms (Lamont et al., 2022). The role of oral microbiota in cancer pathogenesis has been elucidated through three proposed mechanisms: the induction of chronic inflammatory mediators, direct effects on the cell cycle, and the production of certain carcinogens (Lamont et al., 2022; Zhang et al., 2018). Numerous studies have demonstrated that bacteria associated with periodontal disease, particularly *P. gingivalis*, are capable of increasing the risk of developing cancers of the oral cavity and gastrointestinal tract (Lamont et al., 2022). Existing evidence indicates that *P. gingivalis* contributes to the progression of oral squamous cell carcinoma (OSCC), esophageal cancer, and pancreatic cancer by facilitating epithelial-mesenchymal transition (EMT), inhibiting apoptosis of epithelial cells, and promoting immune evasion (Lamont et al., 2022; Zhang et al., 2018). Desmocollin-2 (DSC2), a member of the calreticulin protein family, serves as a crucial component of the bridging granule, primarily responsible for maintaining intercellular adhesion. A reduction in DSC2 expression has been correlated with a more aggressive cancer phenotype (Xin et al., 2014). Research has shown that *P. g*-OMVs can inhibit DSC2 expression by targeting DSC2 binding through the small RNA sRNA23392 carried by *P. g*-OMVs, thereby promoting the invasion and migration of the OSCC cell line HSC-3 (Liu et al., 2021). These findings suggest that *P. g*-OMVs play a role in cancer development; however, research in this domain remains limited. Furthermore, it has been demonstrated that OMVs derived from *H. pylori*, *E. coli*, and *F. nucleatum* can enhance the release of pro-inflammatory cytokines, activate relevant signaling pathways associated with cancer development, and serve as potential biomarkers for cancer detection and prognosis (Park et al., 2021). *P. g*-OMVs exhibit a greater capacity to promote the release of pro-inflammatory cytokines compared to their parental bacteria. Nonetheless, whether they activate a spectrum of signaling pathways that induce cancer development or function as markers for specific cancer detection or prognosis warrants further investigation.

### 5.7 Adverse pregnancy

Adverse pregnancy outcomes (APOs) refer to unexplained abnormalities in embryonic development during gestation, which encompass conditions such as preterm labor, premature rupture of membranes, preeclampsia, miscarriage, intrauterine growth restriction, and low birth weight deliveries (Raju and Berens, 2021). The placenta serves as the primary organ for material exchange between the mother and the fetus, and the proper formation and maintenance of placental homeostasis are critical for healthy fetal development. Trophoblast cells play a pivotal role in both placental formation and the maintenance of homeostasis. Specifically, trophoblast cells can differentiate into invasive extravillous trophoblast (EVT) cells, which migrate and invade the decidua to facilitate maternal vascular remodeling, a crucial step in normal placental development (Knöfler et al., 2019). Concurrently, invading trophoblast cells recruit neutrophils and modulate their function toward anti-inflammatory and reparative phenotypes, thereby sustaining placental immune homeostasis. Deficiencies in the invasive capacity of trophoblast cells, impaired vascular remodeling, and dysregulation of placental immune homeostasis contribute to various pregnancy complications, including preeclampsia, intrauterine growth restriction, and preterm labor (Calo et al., 2017; Calo et al., 2020). Recent studies have demonstrated that *P. g*-OMVs are internalized by trophoblast cells, leading to alterations in the activity and expression of their antioxidant and functional markers. This includes a disruption of their antioxidant capacity and a reduction in the expression of IL-8, IL-6, placental growth factor (PlGF), and vascular endothelial growth factor (VEGF). Furthermore, elevated levels of soluble fms-like tyrosine kinase 1 (sFlt-1) and PlGF, along with an increased sFlt-1/PlGF ratio, have been associated with preeclampsia, serving as supportive indicators for its clinical diagnosis. Additionally, *P. g*-OMVs may exert a pathogenic influence by disrupting interactions between trophoblasts, vascular endothelial cells, and immune cells. Lara et al. (2023) reported that conditioned medium from trophoblast cells pretreated with *P. g*-OMVs resulted in enhanced neutrophil chemotaxis, activation of pro-inflammatory phenotypes, and inhibition of endothelial cell migration. Similarly, Lara et al. (2023) found that *P. g*-OMVs inhibited the glycolytic pathway in human trophoblasts, thereby reducing cell migration and invasive capacity, which ultimately leads to restrictions in placental and fetal development.

### 5.8 Diseases of the respiratory system

Oral microorganisms are recognized as a significant source of lung microbiota and are closely linked to the pathogenesis of various respiratory diseases, including pneumonia, chronic obstructive pulmonary disease, and lung cancer (Maddi et al., 2019). The epithelial cells lining the airways and alveoli form a protective barrier through the adhesion of tight junction proteins, which effectively prevents the invasion of pathogenic microorganisms (Hirakata et al., 2010). Disruption of these tight junction proteins is a primary characteristic of functional impairment in the lung epithelial barrier. During episodes of lung inflammation, the compromised epithelial barrier function results in increased permeability of both the alveolar and airway epithelial barriers, leading to the accumulation of substantial exudate. This process can subsequently cause alveolar edema, acute lung injury, and respiratory distress syndrome (Wittekindt, 2017). He et al. (2020) demonstrated that *P. g*-OMVs can inhibit microbial invasion of the lungs by reducing the availability of adherence sites within the lung epithelial tight junction proteins, specifically affecting the levels of occludin and Claudin-1, thereby disrupting the epithelial barrier. Furthermore, it was observed that *P. g*-OMVs negatively impacted lung epithelial cell viability and induced apoptosis. Consequently, *P. g*-OMVs may play a significant role in the development of respiratory diseases; however, further investigation is required to elucidate the associated pathogenic mechanisms.

## 6 Discussion and prospects

Recent research has increasingly demonstrated that OMVs possess significant biological importance across several critical domains, including bacterial growth, intercellular communication, biofilm formation, bacterial invasion processes, and the modulation of host defense mechanisms. Specifically, *P. g*-OMVs, which are nanoscale spherical structures originating from the outer membrane of *P. gingivalis*, have been shown to invade and disrupt host cellular functions. This disruption occurs through the regulation of plaque biofilm formation and bacterial invasiveness, as well as the induction of host immune dysregulation, leading to periodontal tissue destruction. With the gradual increase of periodontal tissue destruction, *P. g*-OMVs will enter the blood circulation from the ulcerated periodontal pocket site or disseminate to distant organs by means of the nerve pathway. Once *P. g*-OMVs reach the distant organs, they will affect the occurrence and development of systemic diseases through a series of complex mechanisms. On the one hand, *P. g*-OMVs can induce a sustained, low-grade inflammatory response, resulting in a long-term chronic inflammatory state; on the other hand, they also affect and destroy the subcellular structure of host cells and various bioactive substances, seriously interfering with the normal metabolism and physiological functions of cells. More importantly, *P. g*-OMVs can promote pro-inflammatory programmed cell death, which not only interferes with the normal cellular metabolic pathways but also induces inflammatory reactions, ultimately leading to the destruction of periodontal tissue.

The current understanding of the physiological and pathological effects of *P. g*-OMVs is expanding; however, numerous research gaps remain to be addressed. For instance, a comprehensive analysis of the specific components of *P. g*-OMVs under varying environmental conditions is still lacking. The changes in the composition of *P. g*-OMVs, the interactions among these components, and their effects on pathogenicity have not been fully elucidated. Future research should employ high-resolution mass spectrometry and multi-omics techniques to conduct a thorough analysis of the proteins, lipids, and metabolites present in *P. g*-OMVs. This approach will help to reveal the diversity and complexity of their compositions. Additionally, transcriptome sequencing (RNA-seq) and other methodologies should be utilized to investigate the gene expression profiles of *P. g*-OMVs under different environmental conditions, thereby enhancing our understanding of the dynamics of their compositions and their impact on pathogenicity. Moreover, the pathogenic role of *P. g*-OMVs and the underlying mechanisms require more in-depth investigation. Although existing studies have indicated that *P. g*-OMVs influence the occurrence and development of diseases through multiple complex pathways, the specific interactions among these pathways and their synergistic effects leading to periodontal tissue destruction and the onset of systemic diseases remain inadequately explored. Future research should incorporate gene editing techniques to investigate the intricate networks and interactions of *P. g*-OMVs and how these pathways collectively contribute to inflammatory responses and tissue damage. The interactions between *P. g*-OMVs and other pathogens also warrant further examination. While it has been established that *P. g*-OMVs can aggregate various oral pathogens, thereby facilitating plaque biofilm formation, the nature of the interactions between *P. g*-OMVs and these oral pathogens, as well as their OMVs, and the subsequent effects of such interactions on host cells have not been thoroughly investigated. Future studies should employ techniques such as multispecies co-culture, macrogenomics, and macrotranscriptomics to gain deeper insights into the interactions between *P. g*-OMVs and other pathogens and their implications for disease progression. *P. g*-OMVs have been shown to facilitate the transfer of human immunodeficiency virus (HIV) from mucosal surfaces to subcutaneous tissues, as well as to interact with a diverse array of immune cells. This discovery offers novel insights into the interactions between bacterial and viral infections and may have significant implications for the development of new therapeutic strategies (Dong et al., 2018). Future research should further investigate the dynamics of bacterial-viral interactions to enhance our understanding of the pathogenic mechanisms underlying diseases and to formulate more effective therapeutic interventions. Furthermore, there exist numerous blind spots and uncertainties in the association studies concerning *P. g*-OMVs and systemic diseases. At present, research on the relationship between *P. g*-OMVs and systemic diseases, aside from CVD and AD, is limited. Additionally, there is a deficiency of clinical evidence to substantiate the association between *P. g*-OMVs and indicators of systemic diseases. Moving forward, it is imperative to prioritize multicenter clinical studies and employ cellular and animal models to thoroughly investigate the mechanisms by which *P. g*-OMVs influence systemic diseases.

In summary, a comprehensive and systematic investigation of the composition, pathogenic mechanisms, interactions with other pathogens, and associations with systemic diseases of *P. g*-OMVs will not only elucidate their precise roles in disease pathogenesis but also offer novel insights into future therapeutic strategies. These strategies may include the development of specific antibodies or vaccines aimed at inhibiting the role of *P. g*-OMVs in periodontitis; the application of nanotechnology or drug delivery systems to directly target anti-inflammatory drugs or immunomodulators to *P. g*-OMVs or the cells affected by them, thereby mitigating inflammation and cellular damage; and the reduction of *P. gingivalis* and *P. g*-OMVs through enhanced oral hygiene practices, decreased plaque formation, and the utilization of antimicrobial agents. Such advancements are expected to pave the way for a new era in the prevention and treatment of periodontitis and its associated systemic diseases.
